# 2-Chloro-*N*-(4-meth­oxy­phen­yl)benzamide

**DOI:** 10.1107/S1600536810043035

**Published:** 2010-10-30

**Authors:** Aamer Saeed, Jim Simpson

**Affiliations:** a Department of Chemistry, Quaid-i-Azam University, Islamabad 45320, Pakistan; b Department of Chemistry, University of Otago, PO Box 56, Dunedin, New Zealand

## Abstract

In the title compound, C_14_H_12_ClNO_2_, the chloro- and meth­oxy-substituted benzene rings are close to orthogonal [dihedral angle = 79.20 (3)°]. These rings also make angles of 45.9 (3) and 33.5 (3)° with the amide –CONH– unit. The meth­oxy substituent lies close to the meth­oxy­benzene ring plane, with a maximum deviation of 0.142 (3) Å for the methyl C atom. The N—H bond is *anti* to the 2-chloro substituent of the aniline ring. In the crystal structure, inter­molecular N—H⋯O hydrogen bonds form *C*(4) chains augmented by a weak C—H⋯O inter­action involving an *ortho* H atom of the meth­oxy benzene ring that generates an *R*_2_^1^(6) motif. The chains stack the mol­ecules into columns down the *b* axis. Adjacent columns are linked by additional C—H⋯O and C—H⋯π contacts, generating a three-dimensional network.

## Related literature

For background to the biological activity of *N*-substituted benzamides and their use in synthesis, see: Saeed *et al.* (2010 ▶). For related structures, see: Saeed *et al.* (2008*a* ▶,*b* ▶, 2009*a* ▶,*b* ▶,*c* ▶). For hydrogen-bond motifs, see: Bernstein *et al.* (1995 ▶). For reference bond length data, see: Allen *et al.* (1987 ▶).
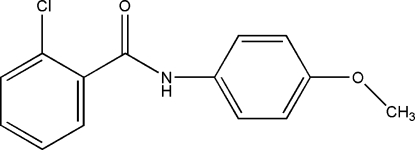


## Experimental

### 

#### Crystal data

C_14_H_12_ClNO_2_
*M* *_r_* = 261.70
Monoclinic, 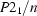

*a* = 13.1819 (10) Å
*b* = 5.0823 (4) Å
*c* = 18.4477 (14) Å
β = 99.563 (3)°
*V* = 1218.72 (16) Å^3^
*Z* = 4
Mo *K*α radiation
μ = 0.31 mm^−1^
*T* = 90 K
0.50 × 0.23 × 0.08 mm

#### Data collection

Bruker APEXII CCD diffractometer
Absorption correction: multi-scan (*SADABS*; Bruker, 2006 ▶) *T*_min_ = 0.885, *T*_max_ = 1.000
21245 measured reflections
4228 independent reflections
3106 reflections with *I* > 2σ(*I*)
*R*_int_ = 0.060

#### Refinement

*R*[*F* ^2^ > 2σ(*F* ^2^)] = 0.041
*wR*(*F* ^2^) = 0.104
*S* = 1.06
4228 reflections
167 parameters
H atoms treated by a mixture of independent and constrained refinement
Δρ_max_ = 0.46 e Å^−3^
Δρ_min_ = −0.28 e Å^−3^

### 

Data collection: *APEX2* (Bruker 2006 ▶); cell refinement: *APEX2* and *SAINT* (Bruker 2006 ▶); data reduction: *SAINT*; program(s) used to solve structure: *SHELXS97* (Sheldrick, 2008 ▶); program(s) used to refine structure: *SHELXL97* (Sheldrick, 2008 ▶) and *TITAN2000* (Hunter & Simpson, 1999 ▶); molecular graphics: *SHELXTL* and *Mercury* (Macrae *et al.*, 2008 ▶); software used to prepare material for publication: *SHELXL97*, *enCIFer* (Allen *et al.*, 2004 ▶), *PLATON* (Spek, 2009 ▶) and *publCIF* (Westrip, 2010 ▶).

## Supplementary Material

Crystal structure: contains datablocks global, I. DOI: 10.1107/S1600536810043035/hg2733sup1.cif

Structure factors: contains datablocks I. DOI: 10.1107/S1600536810043035/hg2733Isup2.hkl

Additional supplementary materials:  crystallographic information; 3D view; checkCIF report

## Figures and Tables

**Table 1:** Hydrogen-bond geometry (Å, °) *Cg*2 is the centroid of the C8–C13 benzene ring.

*D*—H⋯*A*	*D*—H	H⋯*A*	*D*⋯*A*	*D*—H⋯*A*
N1—H1⋯O1^i^	0.854 (17)	2.067 (17)	2.8706 (15)	156.4 (15)
C13—H13⋯O1^i^	0.95	2.69	3.2347 (16)	117
C6—H6⋯O2^ii^	0.95	2.71	3.5657 (17)	150
C12—H12⋯*Cg*2^iii^	0.95	2.88	3.6203 (15)	136

Symmetry codes: (i) 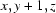; (ii) 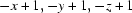; (iii) 
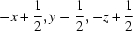
.

**Fig. 1.**
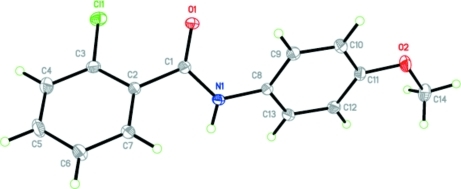
The structure of (I) with displacement ellipsoids for the non-hydrogen atoms drawn at the 50% probability level.

**Fig. 2.**
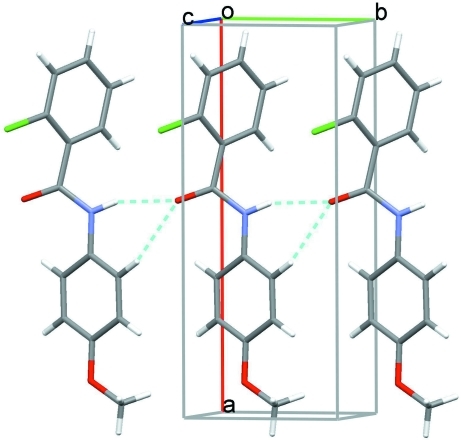
Chains of molecules formed along the b axis with hydrogen bonds drawn as dashed lines.

**Fig. 3.**
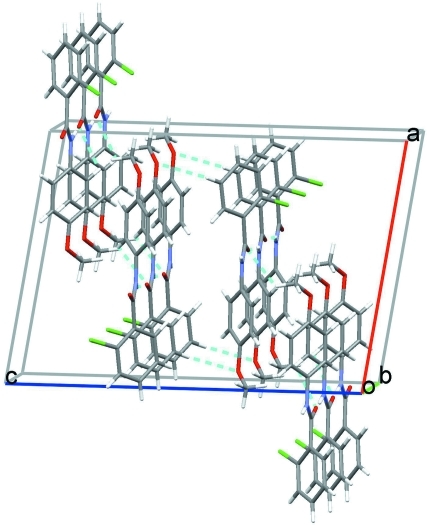
Crystal packing for (I) viewed down the b axis with hydrogen bonds drawn as dashed lines.

## supplementary crystallographic information

### Comment

N-substituted benzamides have numerous pharmaceutical and synthetic applications (Saeed *et al.*, 2010). In the title compound, C_14_H_12_ClNO_2_ (I), the C2···C7,chloro and C8···C13, methoxy substituted benzene rings are close to orthogonal, dihedral angle 79.20 (3)°. These rings also make angles of 45.9 (3) and 33.5 (3)° to the amide –C1(=O1)-N1H1- unit. The methoxy substituent lies close to the C8···C13 ring plane with a maximum deviation of 0.142 (3) Å for C14. Bond distances in the molecule are normal (Allen *et al.*, 1987) and very similar to those other chlorophenylbenzamide derivatives (Saeed *et al.*, 2008*a*, 2009*c*). The N1–H1 bond is *anti* to the Cl1 substituent of the aniline ring in sharp contrast to the *syn* arrangement found in a number of comparable 2-fluoro-benzamide derivatives (Saeed *et al.*, 2008*b*, 2009*a*) including the directly analogous 2-fluoro-*N*-(4-methoxyphenyl)benzamide (Saeed *et al.*, 2009*b*).

In the crystal structure intermolecular N1—H1···O1 hydrogen bonds form C(4) chains (Bernstein *et al.*, 1995) strengthened by a C13—H13···O1 interaction that generates an *R*^1^_2_(6) ring motif. These chains stack the molecules into columns down the *b* axis. The columns are linked by additional C6–H6···O2 and C12—H12···*Cg*2 contacts (*Cg*2 is the centroid of the C8···C13 benzene ring) to generate a three dimensional network.

### Experimental

2-Chlorobenzoyl chloride (1 mmol) in CHCl_3_ was treated with 4-methoxyaniline (3.5 mmol) under a nitrogen atmosphere at reflux for 3 h. Upon cooling, the reaction mixture was diluted with CHCl_3_ and washed consecutively with 1 *M* aq HCl and saturated aq NaHCO_3_. The organic layer was dried over anhydrous sodium sulfate and concentrated under reduced pressure. Crystallization of the residue in ethanol afforded the title compound (80%) as colourless crystals: Anal. calcd. for C_14_H_12_Cl_N_O_2_: C, 64.25; H, 4.62; N, 5.35; found: C, 64.02; H, 4.61; N, 6.23%.

### Refinement

The H1 atom bound to N1 was located in a difference map and refined isotropically. All other H-atoms were positioned geometrically and refined using a riding model with d(C—H) = 0.95 Å, *U*_iso_ = 1.2*U*_eq_ (C) for aromatic and 0.98 Å, *U*_iso_ = 1.5*U*_eq_ (C) for the CH_3_ atoms.

### Crystal data

**Table:**

C_14_H_12_ClNO_2_	*F*(000) = 544
*M**_r_* = 261.70	*D*_x_ = 1.426 Mg m^−^^3^
Monoclinic, *P*2_1_/*n*	Mo *K*α radiation, λ = 0.71073 Å
Hall symbol: -P 2yn	Cell parameters from 3287 reflections
*a* = 13.1819 (10) Å	θ = 2.2–31.3°
*b* = 5.0823 (4) Å	µ = 0.31 mm^−^^1^
*c* = 18.4477 (14) Å	*T* = 90 K
β = 99.563 (3)°	Rectangular block, colourless
*V* = 1218.72 (16) Å^3^	0.50 × 0.23 × 0.08 mm
*Z* = 4	

### Data collection

**Table:**

Bruker APEXII CCD diffractometer	4228 independent reflections
Radiation source: fine-focus sealed tube	3106 reflections with *I* > 2σ(*I*)
graphite	*R*_int_ = 0.060
ω scans	θ_max_ = 32.2°, θ_min_ = 3.5°
Absorption correction: multi-scan (*SADABS*; Bruker, 2006)	*h* = −19→17
*T*_min_ = 0.885, *T*_max_ = 1.000	*k* = −7→7
21245 measured reflections	*l* = −27→27

### Refinement

**Table:**

Refinement on *F*^2^	Primary atom site location: structure-invariant direct methods
Least-squares matrix: full	Secondary atom site location: difference Fourier map
*R*[*F*^2^ > 2σ(*F*^2^)] = 0.041	Hydrogen site location: inferred from neighbouring sites
*wR*(*F*^2^) = 0.104	H atoms treated by a mixture of independent and constrained refinement
*S* = 1.06	*w* = 1/[σ^2^(*F*_o_^2^) + (0.0437*P*)^2^ + 0.2831*P*] where *P* = (*F*_o_^2^ + 2*F*_c_^2^)/3
4228 reflections	(Δ/σ)_max_ = 0.001
167 parameters	Δρ_max_ = 0.46 e Å^−^^3^
0 restraints	Δρ_min_ = −0.28 e Å^−^^3^

### Special details

**Table:**

Geometry. All e.s.d.'s (except the e.s.d. in the dihedral angle between two l.s. planes) are estimated using the full covariance matrix. The cell e.s.d.'s are taken into account individually in the estimation of e.s.d.'s in distances, angles and torsion angles; correlations between e.s.d.'s in cell parameters are only used when they are defined by crystal symmetry. An approximate (isotropic) treatment of cell e.s.d.'s is used for estimating e.s.d.'s involving l.s. planes.
Refinement. Refinement of *F*^2^ against ALL reflections. The weighted *R*-factor *wR* and goodness of fit *S* are based on *F*^2^, conventional *R*-factors *R* are based on *F*, with *F* set to zero for negative *F*^2^. The threshold expression of *F*^2^ > σ(*F*^2^) is used only for calculating *R*-factors(gt) *etc*. and is not relevant to the choice of reflections for refinement. *R*-factors based on *F*^2^ are statistically about twice as large as those based on *F*, and *R*- factors based on ALL data will be even larger.

### Fractional atomic coordinates and isotropic or equivalent isotropic displacement parameters (Å^2^)

**Table:**

	*x*	*y*	*z*	*U*_iso_*/*U*_eq_	
N1	0.48415 (8)	0.3074 (2)	0.61366 (6)	0.0145 (2)	
H1	0.4587 (12)	0.461 (3)	0.6155 (9)	0.017*	
C1	0.42206 (10)	0.0944 (2)	0.60396 (7)	0.0137 (2)	
C12	0.75384 (10)	0.5034 (3)	0.68248 (7)	0.0155 (3)	
H12	0.7893	0.6365	0.7128	0.019*	
C2	0.30910 (9)	0.1564 (2)	0.58872 (7)	0.0127 (2)	
C3	0.23760 (10)	0.0166 (2)	0.62149 (7)	0.0150 (2)	
Cl1	0.27487 (3)	−0.22099 (7)	0.688318 (18)	0.02032 (9)	
C4	0.13309 (11)	0.0697 (3)	0.60386 (8)	0.0212 (3)	
H4	0.0853	−0.0258	0.6270	0.025*	
C5	0.09866 (11)	0.2618 (3)	0.55254 (8)	0.0219 (3)	
H5	0.0271	0.2958	0.5397	0.026*	
C6	0.16843 (10)	0.4055 (3)	0.51967 (7)	0.0180 (3)	
H6	0.1448	0.5388	0.4848	0.022*	
C7	0.27285 (10)	0.3531 (3)	0.53803 (7)	0.0148 (2)	
H7	0.3205	0.4525	0.5158	0.018*	
C8	0.59378 (9)	0.3016 (2)	0.62596 (7)	0.0131 (2)	
C9	0.64888 (10)	0.1142 (3)	0.59279 (7)	0.0154 (2)	
H9	0.6135	−0.0180	0.5620	0.019*	
C10	0.75521 (10)	0.1228 (3)	0.60506 (7)	0.0167 (3)	
H10	0.7927	−0.0051	0.5828	0.020*	
C11	0.80807 (10)	0.3166 (3)	0.64961 (7)	0.0145 (2)	
O2	0.91341 (7)	0.3080 (2)	0.65680 (5)	0.0186 (2)	
C14	0.96893 (10)	0.5186 (3)	0.69682 (8)	0.0222 (3)	
H14A	0.9452	0.6869	0.6742	0.033*	
H14B	1.0426	0.4979	0.6958	0.033*	
H14C	0.9571	0.5153	0.7479	0.033*	
O1	0.45382 (7)	−0.13338 (18)	0.60577 (6)	0.0192 (2)	
C13	0.64644 (10)	0.4933 (3)	0.67043 (7)	0.0159 (3)	
H13	0.6090	0.6201	0.6931	0.019*	

### Atomic displacement parameters (Å^2^)

**Table:**

	*U*^11^	*U*^22^	*U*^33^	*U*^12^	*U*^13^	*U*^23^
N1	0.0114 (5)	0.0092 (5)	0.0221 (6)	0.0006 (4)	0.0001 (4)	−0.0005 (4)
C1	0.0123 (6)	0.0127 (5)	0.0155 (6)	0.0003 (5)	0.0003 (5)	0.0006 (4)
C12	0.0146 (6)	0.0135 (6)	0.0176 (6)	−0.0015 (5)	0.0002 (5)	−0.0013 (5)
C2	0.0117 (6)	0.0117 (5)	0.0144 (6)	−0.0002 (4)	0.0012 (4)	−0.0018 (4)
C3	0.0166 (6)	0.0118 (5)	0.0164 (6)	0.0003 (5)	0.0022 (5)	0.0008 (4)
Cl1	0.02428 (18)	0.01703 (15)	0.01980 (16)	0.00019 (13)	0.00408 (13)	0.00509 (12)
C4	0.0152 (6)	0.0225 (7)	0.0271 (7)	−0.0008 (5)	0.0073 (5)	0.0032 (6)
C5	0.0107 (6)	0.0273 (7)	0.0278 (7)	0.0032 (5)	0.0035 (5)	0.0034 (6)
C6	0.0148 (6)	0.0211 (7)	0.0179 (6)	0.0040 (5)	0.0023 (5)	0.0027 (5)
C7	0.0139 (6)	0.0144 (6)	0.0164 (6)	0.0017 (5)	0.0030 (5)	0.0009 (4)
C8	0.0108 (5)	0.0115 (5)	0.0166 (6)	0.0001 (5)	0.0014 (4)	0.0016 (4)
C9	0.0151 (6)	0.0139 (6)	0.0172 (6)	−0.0008 (5)	0.0023 (5)	−0.0021 (5)
C10	0.0157 (6)	0.0160 (6)	0.0194 (6)	0.0011 (5)	0.0058 (5)	−0.0014 (5)
C11	0.0117 (6)	0.0167 (6)	0.0150 (6)	−0.0007 (5)	0.0019 (4)	0.0027 (5)
O2	0.0105 (4)	0.0234 (5)	0.0219 (5)	−0.0011 (4)	0.0027 (4)	−0.0029 (4)
C14	0.0129 (6)	0.0278 (7)	0.0249 (7)	−0.0058 (5)	0.0001 (5)	−0.0021 (6)
O1	0.0135 (5)	0.0103 (4)	0.0329 (6)	0.0012 (4)	0.0012 (4)	0.0007 (4)
C13	0.0135 (6)	0.0124 (6)	0.0214 (6)	0.0010 (5)	0.0020 (5)	−0.0007 (5)

### Geometric parameters (Å, °)

**Table:**

N1—C1	1.3506 (17)	C5—H5	0.9500
N1—C8	1.4255 (16)	C6—C7	1.3876 (18)
N1—H1	0.854 (17)	C6—H6	0.9500
C1—O1	1.2298 (15)	C7—H7	0.9500
C1—C2	1.5021 (17)	C8—C13	1.3841 (18)
C12—C11	1.3870 (18)	C8—C9	1.3985 (17)
C12—C13	1.3970 (18)	C9—C10	1.3829 (18)
C12—H12	0.9500	C9—H9	0.9500
C2—C3	1.3957 (18)	C10—C11	1.3931 (18)
C2—C7	1.3977 (17)	C10—H10	0.9500
C3—C4	1.3883 (19)	C11—O2	1.3734 (15)
C3—Cl1	1.7373 (13)	O2—C14	1.4305 (17)
Cl1—O1	3.0456 (11)	C14—H14A	0.9800
C4—C5	1.383 (2)	C14—H14B	0.9800
C4—H4	0.9500	C14—H14C	0.9800
C5—C6	1.3901 (19)	C13—H13	0.9500
			
C1—N1—C8	125.48 (11)	C6—C7—H7	119.5
C1—N1—H1	120.4 (11)	C2—C7—H7	119.5
C8—N1—H1	114.0 (11)	C13—C8—C9	119.53 (12)
O1—C1—N1	123.69 (12)	C13—C8—N1	118.37 (11)
O1—C1—C2	121.67 (11)	C9—C8—N1	122.07 (12)
N1—C1—C2	114.63 (11)	C10—C9—C8	119.53 (12)
C11—C12—C13	119.19 (12)	C10—C9—H9	120.2
C11—C12—H12	120.4	C8—C9—H9	120.2
C13—C12—H12	120.4	C9—C10—C11	120.85 (12)
C3—C2—C7	118.26 (11)	C9—C10—H10	119.6
C3—C2—C1	122.13 (11)	C11—C10—H10	119.6
C7—C2—C1	119.56 (11)	O2—C11—C12	124.46 (12)
C4—C3—C2	120.99 (12)	O2—C11—C10	115.67 (11)
C4—C3—Cl1	116.99 (10)	C12—C11—C10	119.87 (12)
C2—C3—Cl1	121.97 (10)	C11—O2—C14	116.64 (10)
C3—Cl1—O1	72.23 (5)	O2—C14—H14A	109.5
C5—C4—C3	119.83 (13)	O2—C14—H14B	109.5
C5—C4—H4	120.1	H14A—C14—H14B	109.5
C3—C4—H4	120.1	O2—C14—H14C	109.5
C4—C5—C6	120.26 (13)	H14A—C14—H14C	109.5
C4—C5—H5	119.9	H14B—C14—H14C	109.5
C6—C5—H5	119.9	C1—O1—Cl1	82.11 (8)
C7—C6—C5	119.61 (13)	C8—C13—C12	121.01 (12)
C7—C6—H6	120.2	C8—C13—H13	119.5
C5—C6—H6	120.2	C12—C13—H13	119.5
C6—C7—C2	121.04 (12)		

### Hydrogen-bond geometry (Å, °)

**Table:**

Cg2 is the centroid of the C8–C13 benzene ring.

**Table:**

*D*—H···*A*	*D*—H	H···*A*	*D*···*A*	*D*—H···*A*
N1—H1···O1^i^	0.854 (17)	2.067 (17)	2.8706 (15)	156.4 (15)
C13—H13···O1^i^	0.95	2.69	3.2347 (16)	117
C6—H6···O2^ii^	0.95	2.71	3.5657 (17)	150
C12—H12···Cg2^iii^	0.95	2.88	3.6203 (15)	136

Symmetry codes: (i) *x*, *y*+1, *z*; (ii) −*x*+1, −*y*+1, −*z*+1; (iii) −*x*+1/2, *y*−1/2, −*z*+1/2.
